# *Lactobacillus* *paragasseri* BBM171 Ameliorates Allergic Airway Inflammation Induced by Ovalbumin in Mice via Modulating the Th1/Th2 Balance

**DOI:** 10.3390/microorganisms10102041

**Published:** 2022-10-15

**Authors:** Shih-Hsuan Cheng, Tzu-Ying Yang, Chih-Chieh Hsu, Yu-Hsuan Wei, Chien-Chen Wu, Ying-Chieh Tsai

**Affiliations:** 1 Bened Biomedical Co., Ltd., Taipei 10448, Taiwan; 2 Institute of Biochemistry and Molecular Biology, National Yang Ming Chiao Tung University, Taipei 11221, Taiwan

**Keywords:** *Lactobacillus paragasseri* BBM171, allergy, ovalbumin, T helper cells

## Abstract

Supplementation with specific probiotics has been shown to improve allergic airway symptoms. This study aimed to investigate immunomodulatory effects of a potential probiotic strain isolated from breast milk, *Lactobacillus* *paragasseri* BBM171 (BBM171), in an ovalbumin (OVA)-induced allergic mouse model. OVA-sensitized and OVA-challenged BALB/c mice were orally administered live or heat-inactivated BBM171 for 48 consecutive days. After the last allergen challenge, serum immunoglobulin (Ig) levels, inflammatory cell levels in the lungs, and cytokine levels in bronchoalveolar lavage fluid (BALF) were assessed. The results showed that oral administration of live or heat-inactivated BBM171 decreased serum levels of total IgE, OVA-specific IgE, and OVA-specific IgG1, while increasing OVA-specific IgG2a and reducing the extent of airway inflammation in OVA-induced allergic mice. In addition, both live and heat-inactivated BBM171 modulated the cytokine profile in BALF to a type 1 T helper (Th1) response. Furthermore, ex vivo experiments using OVA-induced allergic mouse splenocytes showed that both live and heat-inactivated BBM171 could regulate the Th1/Th2 balance, decrease the proinflammatory cytokine interleukin (IL)-17 level, and increase the anti-inflammatory cytokine IL-10 level. Taken together, these results suggest that oral administration of live or heat-inactivated BBM171 improved allergen-induced airway inflammation symptoms by modulating the host immune response toward Th1 dominance.

## 1. Introduction

Allergic airway disorders, such as allergic rhinitis (AR) and asthma, are chronic airway inflammatory diseases whose incidence continues to rise, making them a major public health problem. The pattern of inflammation in response to experimental allergen challenge is similar in AR and asthma [1]. Therefore, both disease models have been widely used to study allergic airway inflammation. The immune response of type 2 T helper cells (Th2) is thought to be a crucial mechanism involved in allergic inflammation, which involves the secretion of a range of cytokines, including interleukin (IL)-4, IL-5, and IL-13. On the other hand, type 1 T helper cell (Th1)-driven cytokines including interferon-gamma (IFN-γ), IL-2, and IL-12 inhibits the proliferation of Th2 cells [2]. Differential expression of the balanced relationship between Th1 and Th2 in the immune system is important for the development of allergic diseases [3]. In addition, Th17 and regulatory T (Treg) cells have been reported to play a role in the pathogenesis of allergic airway inflammation. Th17 cells are characterized by the secretion of IL-17, which mediate eosinophilic airway inflammation [4]. The suppression of Th2 cell-driven inflammatory responses by Treg cells is crucial for the development of asthma tolerance. Thus, evidence suggests that imbalanced Th1/Th2 and Th17/Treg cell responses play a crucial role in antigen-induced airway inflammation [5,6].

Probiotics are widely used in various commercial food products, some of which are known to improve inflammatory diseases and promote human health [7]. One of the reported health-promoting properties of probiotics is immunomodulation or immune tolerance in allergic diseases [8]. Probiotics interact with intestinal epithelial or immune cells to induce the production of different cytokines through Toll-like receptors [9]. For example, Ren et al. showed that *Lactobacillus salivarius* and *Lactobacillus plantarum* promote naive T-cell polarization to Th1 cells in vitro and in vivo, suggesting their potential to treat allergic diseases by modulating immune responses [10]. In addition, probiotics have been shown to alleviate allergic asthma by preventing the polarization of Th2 and Th17 cells and regulating STAT6 and T-bet transcription factors to increase Th1 response [11]. Another study showed that *Lactobacillus rhamnosus* GG (LGG) improved allergic symptoms in birch pollen-induced allergic asthma by suppressing the levels of Th2 cytokines, IL-13, and IL-5 [12].

Many commensal or beneficial microbes are present in breast milk, which influence the composition of the infant gut microbiota and contribute to the development of innate and acquired immunity [13]. Breastfeeding plays an important role in protecting against allergic diseases in early childhood, and breast milk may be a good source of probiotics [14]. For example, two probiotic strains isolated from breast milk, *L. salivarius* CECT 5713 and *Lactobacillus fermentum* CECT 5716, promote the secretion of Th1 cytokines by macrophages in the absence of immune stimulation. In contrast, the secretion of Th1 cytokines is reduced when immune cells are stimulated by lipopolysaccharide (LPS). Furthermore, both strains stimulate antigen-presenting cells (APCs) to secrete Treg cytokines [15,16].

*Lactobacillus paragasseri* was recently classified as a novel species and a sister taxon of *Lactobacillus gasseri* [17]. *L. gasseri* has gained considerable attention because of its ability to maintain healthy vaginal flora and aid in digestion [18]. In addition, a meta-analysis has indicated that four specific species, including *L. gasseri*, were effective in the treatment of *Helicobacter pylori* infection [19]. However, studies on *L. paragasseri* are scarce. In this study, we aimed to investigate the anti-allergic potential of *L. paragasseri* BBM171 (BBM171), a strain isolated from breast milk in Taiwan, for its immunomodulatory ability to stimulate high levels of IFN-γ production in human peripheral blood mononuclear cells (hPBMCs). The effects of BBM171 on ovalbumin (OVA)-induced allergic mice were evaluated by assessing serum immunoglobulin (Ig) levels, inflammatory cell infiltration, mucus secretion in lung tissue, and bronchoalveolar lavage fluid (BALF) cytokine profiles. In addition, we evaluated the effects of BBM171 on Th1, Th2, Th17, and Treg cytokine production in splenocytes isolated from OVA-sensitized mice. To our knowledge, BBM171 is the first *L. paragasseri* strain that shows anti-allergic potential. These results hold promise for the future development of *L. paragasseri* BBM171 to alleviate allergic symptoms in humans.

## 2. Materials and Methods

### 2.1. Preparation of Lactobacillus paragasseri BBM171

*L. paragasseri* BBM171 (DSM 34311) was isolated from breast milk in Taiwan and routinely cultivated in Man Rogosa Sharpe (MRS; BD, Sparks, MD) broth at 37 °C for 18–20 h. For live *L. paragasseri* BBM171 preparation, pelleted bacteria were washed twice with sterile phosphate-buffered saline (PBS) and resuspended to attain a final concentration of 5 × 10^8^ colony-forming units (CFUs)/mL in PBS before oral administration. For the preparation of heat-inactivated *L. paragasseri* BBM171, the pellet was resuspended to a final concentration of approximately 5 × 10^8^ CFU/mL in PBS, heat-inactivated at 100 °C for 20 min, and stored at −20 °C until use.

### 2.2. Animals

BALB/c mice (female, 6–8 weeks old) were purchased from BioLASCO Taiwan Co., Ltd., Taipei, Taiwan. Food and water were provided to the mice ad libitum. The animal room was kept on a 12 h light—12 h dark cycle. All animal experimental procedure were reviewed and approved by the Institutional Animal Use and Care Committee (IACUC No. 1060606; date of approval: June 6, 2017), National Yang Ming Chiao Tung University.

### 2.3. OVA-Induced Allergic Airway Inflammation in Mice

**Figure 1:**
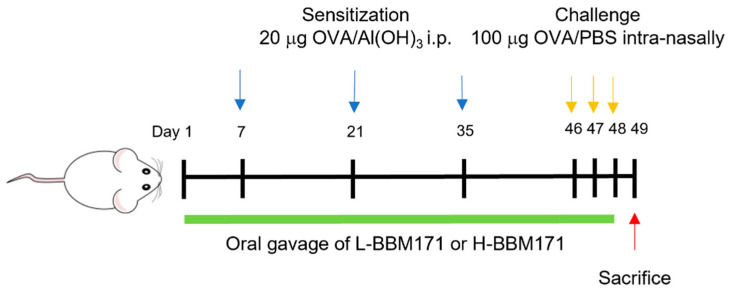
Experimental procedure of ovalbumin (OVA)-induced allergic mouse model. Female BALB/c mice were orally administered live *L. paragasseri* BBM171 (L-BBM171) or heat-inactivated *L. paragasseri* BBM171 (H-BBM171) by gavage for 48 days and injected with 20 µg OVA/Al(OH)_3_ intraperitoneally on days 7, 21, and 35. On days 46, 47, and 48, mice were challenged with 100 µg OVA/PBS by intranasal administration.

An OVA-induced allergic mouse model was performed according to a previous report [20], with some modifications, to assess the effect of BBM171. The experimental procedure is illustrated in Figure 1. Mice were randomly divided into four groups (n = 8 per group): healthy control mice (CON), OVA-induced allergic mice (OVA), allergic mice supplemented with live BBM171 (L-BBM171), and allergic mice supplemented with heat-inactivated BBM171 (H-BBM171). Mice in the OVA, L-BBM171, and H-BBM171 groups were sensitized by intraperitoneal injection with 200 µL aluminum hydroxide (Al(OH)_3_) (Pierce Biotechnology, Rockford, IL, USA) containing 20 µg OVA on days 7, 21, and 35. On days 46, 47, and 48, mice were challenged intranasally with 100 µg OVA solution (in 40 µL PBS) under anesthesia with pentobarbital. The CON group received Al(OH)_3_ only when sensitized and PBS during the challenge period. Mice in the L-BBM171 and H-BBM171 groups were orally administered live BBM171 or heat-inactivated BBM171 in 200 µL PBS (10^8^ CFU/mouse/day) from day 1 to the last challenge day (day 48), respectively. The CON and OVA groups were orally administered 200 µL PBS per day. On day 49, all mice were sacrificed, and BALF and blood samples were collected for further analyses. 

### 2.4. Immunoglobulin Measurements

Commercial enzyme-linked immunosorbent assay (ELISA) kits were used to analyze Igs in the blood, including total IgE (Bethyl Laboratory Inc., Montgomery, TX, USA) and OVA-specific Igs (Alpha Diagnostic International Inc., San Antonio, TX, USA), according to the manufacturer’s instructions.

### 2.5. Histological Analysis 

After sacrifice, lung tissues were fixed in 10% formaldehyde solution. Histopathological staining of lung tissues was performed according to standard procedures. The specimens were then dehydrated using ethyl alcohol and xylene. Tissues were embedded in paraffin and sliced into 5 μm thick sections. The sections were stained with hematoxylin and eosin (H&E) to observe inflammatory cell infiltration around the airway and evaluate the inflammation score {no infiltration (0 score); a little (1 score); more (2 scores); a large number, less than a group (3 scores); a large number of groups (4 scores)}. In addition, the distribution of goblet cells and mucus secretion were quantified using periodic acid-Schiff (PAS) staining. presence of airway goblet cells {no (0 score); <25% (1 score); 25–50% (2 scores); 50–75% (3 scores); >75% (4 scores)} [21].

### 2.6. Analysis of Bronchoalveolar Lavage Fluid (BALF) 

The trachea was exposed, and the airways were lavaged twice with 1 mL of Hanks’ balanced salt solution (HBSS) twice using a tracheal cannula. Cells in BALF were collected by centrifugation (400× *g*, 4 °C, 10 min), and the supernatant was stored at −80 °C for further cytokine analysis. Cell pellets from the BALF were resuspended in HBSS containing 2% fetal bovine serum, and the numbers of lymphocytes and eosinophils were analyzed using a XT-1800iV cytometer (Sysmex, Hyogo, Japan).

### 2.7. Stimulation of Splenocytes Ex Vivo

Mice (n = 4) were sensitized by intraperitoneal injection of 200 µL of Al(OH)_3_ containing 50 µg OVA on days 0 and 14. On day 21, the mice were sacrificed and their splenocytes were isolated, pulled, and adjusted to a concentration of 2 × 10^6^ cells/mL. The collected splenocytes were then cultured with OVA (100 µg/mL) in the presence of live or heat-inactivated BBM171 at 2 × 10^7^ cells for 24 h. The culture medium was collected for cytokine measurements.

### 2.8. Cytokine Measurements

Cytokines, including IL-2, IL-4, IL-5, IL-12, IL-13, IFN-γ, IL-17A, and IL-10, were measured using commercial ELISA kits (R&D Systems, Minneapolis, MN, USA).

### 2.9. Statistical Analysis

Data are expressed as mean ± standard error of the mean (SEM). Differences between means were tested for statistical significance using one-way analysis of variance (ANOVA) followed by Tukey’s posthoc test. Differences between groups were considered statistically significant when the *p* < 0.05.

## 3. Results

### 3.1. Oral Administration of L. paragasseri BBM171 Decreased the Production of Serum Igs in Ova-Induced Allergic Mice

**Figure 2:**
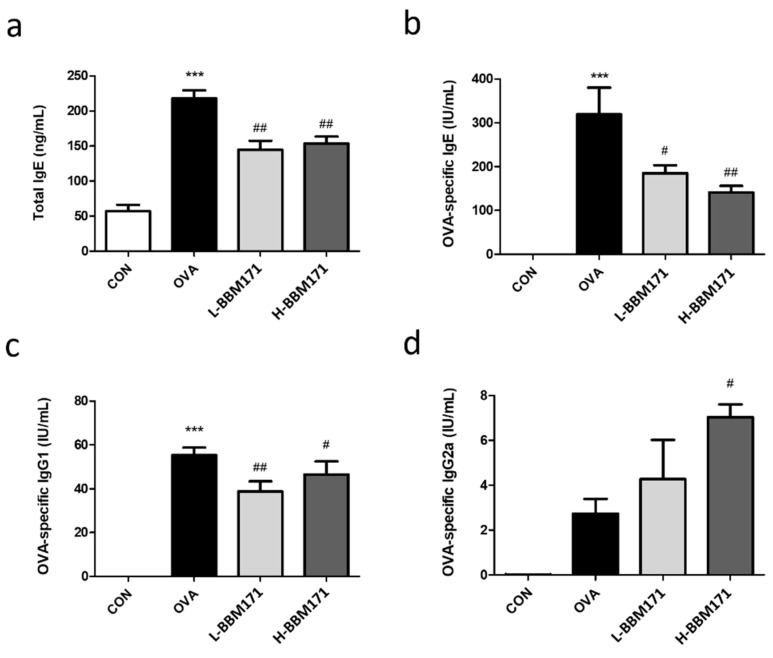
Effects of *L. paragasseri* BBM171 on the serum immunoglobulin levels in OVA-induced allergic mice. (**a**) IgE. (**b**) OVA-specific IgE. (**c**) OVA-specific IgG1. (**d**) OVA-specific IgG2. Data are expressed as mean ± SEM and analyzed by one-way ANOVA with Tukey’s post hoc test. Significant differences at *** *p* < 0.001 compared with the CON group. ^#^ *p* < 0.05 and ^##^ *p* < 0.01 compared with the OVA group.

Total IgE and OVA-specific Igs were assessed to evaluate individual responses to allergen exposure. Serum total IgE levels were significantly increased (*p* < 0.001) in the OVA group compared with the CON group (Figure 2a). L-BBM171 (live BBM171) and H-BBM171 (heat-inactivated BBM171) groups had lower serum total IgE levels than the OVA group (*p* < 0.01) In the CON group, OVA-specific Igs, including IgE, IgG1, and IgG2a, were not detected (Figure 2b–d). Compared with the CON group, the levels of OVA-specific IgE and IgG1 were higher in the OVA group, which were reduced by the supplementation of live or heat-inactivated BBM171 (Figure 2b,c). In addition, mice in the L-BBM171 group had increased serum OVA-specific IgG2 levels compared to those in the OVA group, which was dependent on Th1 cells (Figure 2d). 

### 3.2. L. paragasseri BBM171 Ameliorated Airway Inflammation in Ova-Induced Allergic Mice

**Figure 3:**
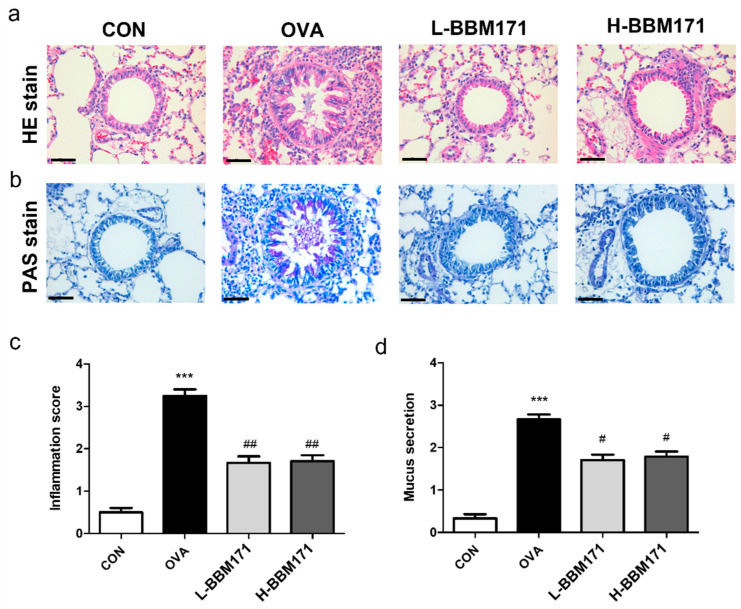
Oral administration of *L. paragasseri* BBM171 reduced inflammatory cell infiltration and the amount of mucus in the lung tissues of OVA-induced allergic mice. (**a**) Hematoxylin and eosin (H&E) staining. (**b**) Periodic acid-Schiff (PAS) staining. (**c**) The inflammation score represents the semi-quantitative results of airway inflammatory cells stained with H&E. (**d**) semi-quantitative results of mucus secretion stained with PAS. Bars = 50 μm. Data are expressed as mean ± SEM and analyzed by one-way ANOVA with Tukey’s post hoc test. Significant differences at *** *p* < 0.001 compared with the CON group. ^#^ *p* < 0.05 and ^##^ *p* < 0.01 compared with the OVA group.

Inflammatory cell infiltration and mucus production in the lung tissues were analyzed to assess the development of airway inflammation. Compared to the CON group, histological analysis and PAS staining of lung sections revealed significant increases in inflammatory cell infiltration (Figure 3a) and mucus production (Figure 3b) in the OVA group. However, these effects were alleviated by the supplementation of live or heat-inactivated BBM171. Semi-quantitative results of airway inflammatory cell infiltration and mucus secretion are shown in Figure 3c,d.

### 3.3. L. paragasseri BBM171 Attenuated the Accumulation of Inflammatory Cells in TheBALF

**Figure 4:**
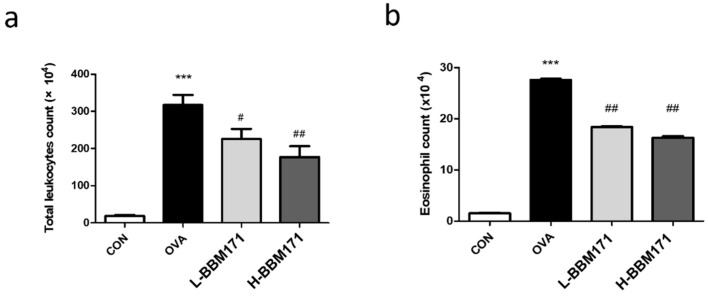
Effects of *L. paragasseri* BBM171 intervention on the distribution of immune cells in BALF. (**a**) total count of leukocytes. (**b**) cell population of eosinophils. Data are expressed as mean ± SEM and analyzed by one-way ANOVA with Tukey’s post hoc test. Significant differences at *** *p* < 0.001 compared with the CON group. ^#^ *p* < 0.05 and ^##^ *p* < 0.01 compared with the OVA group.

To assess the effect of *L. paragasseri* BBM171 on OVA-induced recruitment and accumulation of inflammatory cells in the lungs, the levels of leukocytes and eosinophils in BALF were measured. Compared to the CON group, the levels of total leukocytes (Figure 4a) and eosinophils (Figure 4b) were significantly higher in the OVA group. However, these effects were alleviated by the supplementation of live or heat-inactivated BBM171.

### 3.4. L. paragasseri BBM171 Altered the Th1/Th2 Responses in the Lungs of Ova-Induced Allergic Mice

**Figure 5:**
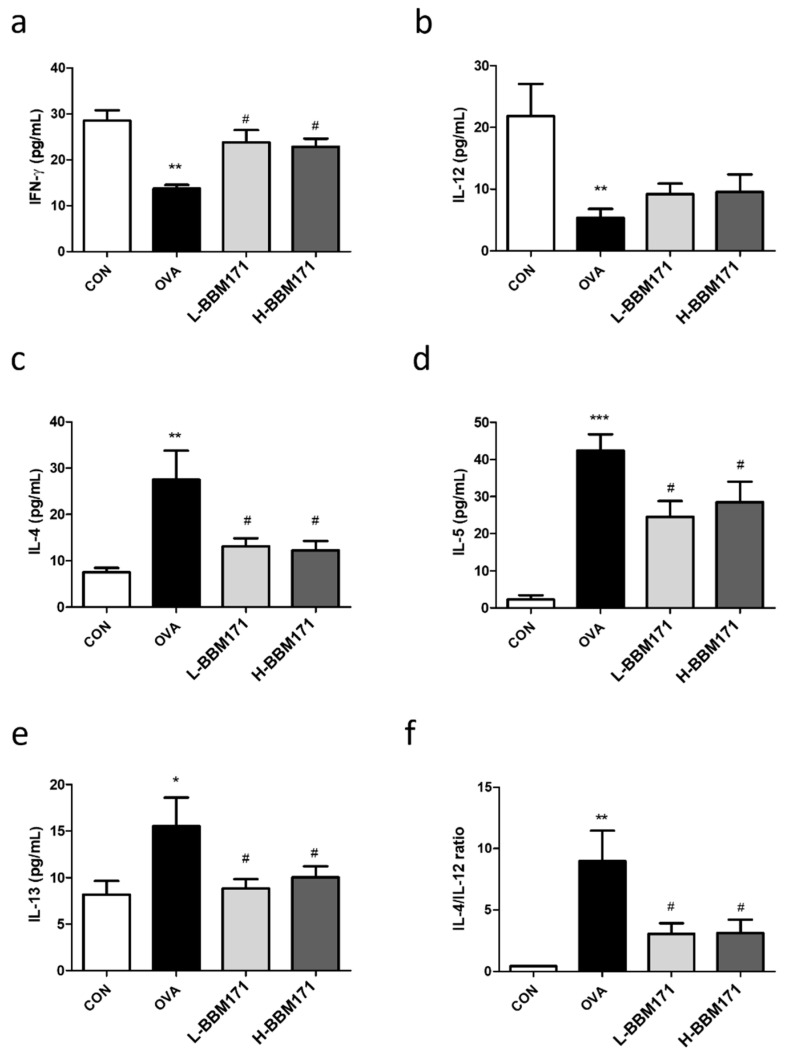
Effects of *L. paragasseri* BBM171 on the production of Th1 and Th2-related cytokines in the BALF determined by ELISA. The concentration of (**a**) IFN-γ. (**b**) IL-12. (**c**) IL-4. (**d**) IL-5. (**e**) IL-13. (**f**) IL-4/IL-12 ratio. Data are expressed as mean ± SEM and analyzed by one-way ANOVA with Tukey’s post hoc test. Significant differences at * *p* < 0.05, ** *p* < 0.01, and *** *p* < 0.001 compared with the CON group. ^#^ *p* < 0.05 compared with the OVA group.

The concentrations of Th1 and Th2 cytokines in BALF were measured. Compared to the CON group, the levels of classic Th1 cytokines (IFN-γ and IL-12) were significantly decreased in the OVA group (Figure 5a,b). Supplementation with live or heat-inactivated BBM171 attenuated the decrease in IFN-γ but not IL-12. In addition, compared to the CON group, the levels of Th2 cytokines (IL-4, IL-5, and IL-13) were significantly elevated in the OVA group (Figure 5c–e), which could be alleviated by live or heat-inactivated BBM171 supplementation. Furthermore, compared to the CON group, the IL-4/IL-12 ratio was significantly higher in the OVA group, suggesting Th2 dominance in the OVA group (Figure 5f). Supplementation with live or heat-inactivated BBM171 significantly decreased the IL-4/IL-12 ratio in the BALF of OVA-induced allergic mice, suggesting that *L. paragasseri* BBM171 regulates the immune response toward Th1.

### 3.5. Effects of L. paragasseri BBM171 on the Cytokine Profile in Splenocytes from OVA-Induced Allergic Mice 

**Figure 6:**
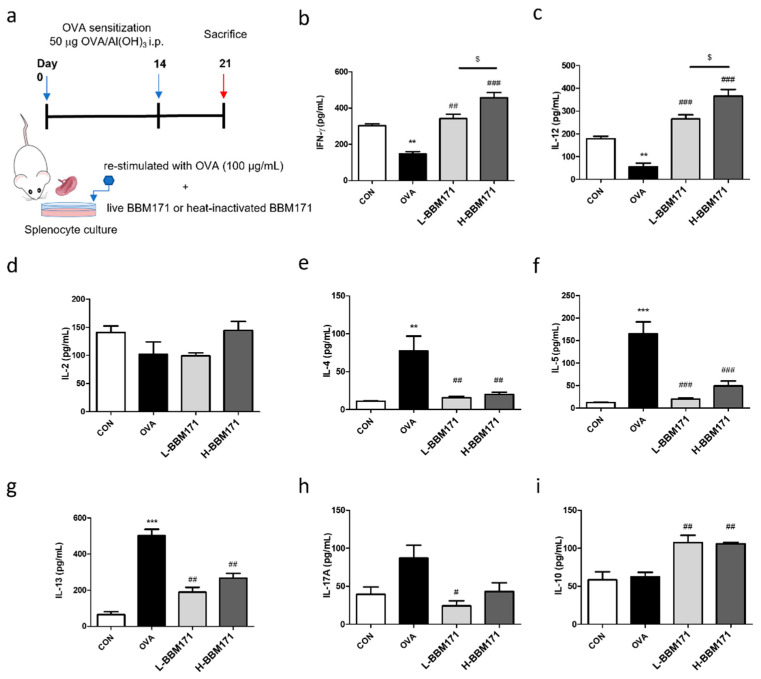
Effects of *L. paragasseri* BBM171 on T-cell cytokines produced by splenocytes in response to OVA from OVA-sensitized mice. (**a**) The ex vivo study procedure. (**b**) IFN-γ. (**c**) IL-12. (**d**) IL-2. (**e**) IL-4. (**f**) IL-5. (**g**) IL-13. (**h**) IL-17A. (**i**) IL-10. Data are expressed as mean ± SEM and analyzed by one-way ANOVA with Tukey’s post hoc test. Significant differences at ** *p* < 0.01 and *** *p* < 0.001 compared with the CON group. ^#^ *p* < 0.05, ^##^ *p* < 0.01, and ^###^ *p* < 0.001 compared with the OVA group. ^$^ *p* < 0.05 between L-BBM171 and H-BBM171 groups.

To further evaluate the immunomodulatory effects of live and heat-inactivated BBM171 on T cell responses, an ex vivo experiment was performed (Figure 6a). Splenocytes from OVA-sensitized mice were isolated, collected, divided into four groups for different treatments, and incubated for 24 h to analyze cytokine levels in the supernatant. Compared to the CON (addition of PBS) group, re-stimulation with OVA reduced the production of Th1-driven IFN-γ (Figure 6b) and IL-12 (Figure 6c), which was significantly increased by the addition of live or heat-inactivated BBM171. In addition, heat-inactivated BBM171 showed more profound effects than live BBM171 (Figure 6b,c). For IL-2, a Th1-driven cytokine, there was no significant difference among the four groups (Figure 6d). Compared with the CON group, re-stimulation with OVA increased the production of Th2-driven cytokines IL-4 (Figure 6e), IL-5 (Figure 6f), and IL-13 (Figure 6g), which was reversed by the addition of live or heat-inactivated BBM171. In addition, the level of the cardinal Th17 cytokine, IL-17A, was significantly lower in the L-BBM171 group than in the OVA group (Figure 6h). Moreover, both live and heat-inactivated BBM171 regulated Treg responses to increase IL-10 production in OVA-treated splenocytes (Figure 6i). These results indicate that *L. paragasseri* BBM171 effectively regulates the Th1/Th2 balance in splenocyte culture in response to OVA challenge, and that *L. paragasseri* BBM171 reduces the levels of proinflammatory cytokine IL-17A while increasing those of the anti-inflammatory cytokine IL-10.

## 4. Discussion

Allergic airway inflammation is an abnormally aggravated reaction in response to various factors such as pollen grains, dust mites, and specific foods [22]. Chronic allergic airway inflammation leads to the development of AR or asthma, both of which are associated with high morbidity [23]. Therefore, there is still a need to develop new therapeutic or ameliorative approaches to allergic airway inflammation. In this study, we investigated the effects of *L. paragasseri* BBM171 on OVA-induced allergic inflammation in a mouse model. Allergic airway inflammation was induced by intraperitoneal sensitization and intratracheal OVA challenge (Figure 1). The model mimics the features of allergic airway inflammation, including excessive mucus secretion, eosinophilic airway inflammation, and unique adaptive immune responses, including Th1, Th2, Th17, and Treg cells.

Gorczynski et al. showed that long-term intranasal exposure to OVA triggers strong antigen-specific serum IgE and IgG1 immunoreactivity [24]. As shown in Figure 2, OVA caused significant increases in serum total IgE, OVA-specific IgE, and OVA-specific IgG1 levels, indicating successful establishment of an allergic mouse model with an activated Th2 response [25]. Simultaneously, increased lung inflammation, mucus secretion, and lymphocyte and eosinophil infiltration in the BALF were observed in the OVA group mice (Figure 3 and Figure 4). Interestingly, both live BBM171 and heat-inactivated BBM171 attenuated OVA-induced inflammatory cell infiltration and mucus production in the lungs. In addition, the numbers of lymphocytes and eosinophils were significantly reduced in both live BBM171 and heat-inactivated BBM171 supplementation groups. Our results suggest that the probiotic BBM171 can improve allergic airway inflammation symptoms.

In the present study, BBM171 promoted IFN-γ (Th1 cytokine) production in an in vitro hPBMCs screening model (data not shown); therefore, the potential anti-allergic activity of BBM171 was investigated. Although probiotics are defined as living microorganisms with health benefits to the host, heat-inactivated probiotic strains are also thought to exhibit some physiological functions. Both live and heat-inactivated BBM171 strains were included in this study. As mentioned above, the results showed that both live and heat-inactivated BBM171 improved allergic airway inflammation symptoms, including suppression of serum Igs, inflammatory status, and cellular infiltration in the lungs. Therefore, we further investigated the effect of live and heat-inactivated BBM171 on immunomodulatory cytokines in the BALF. As shown in Figure 5, supplementation with live or heat-inactivated BBM171 resulted in a significant reduction in the production of Th2 cytokines, including IL-4, IL-5, and IL-13, and enhanced the production of Th1-driven cytokine IFN-γ in the BALF. IFN-γ has been reported to prevent antigen-induced development of airway eosinophilia [26], and the results are consistent with our preliminary data obtained using hPBMCs. In addition, the oral administration of BBM171 reduced the accumulation of airway eosinophils in OVA-induced allergic mice. As for the Th1-related cytokine IL-12, there was no significant difference in IL-12 levels between the BBM171 supplementation groups and the OVA group; however, decreased levels of IL-12 were observed in the OVA group compared with the control group. In contrast, a reversal effect was observed in both live and heat-activated BBM171 groups. IL-12 is released by APCs, plays an important role in Th1/Th2 differentiation, and inhibits IL-4-dependent IgE synthesis [27]. We further evaluated the effect of BBM171 on Th1/Th2 differentiation using IL-4 and IL-12 levels. Strikingly, OVA treatment significantly increased the IL-4/IL-12 ratio, which was significantly lower in both the live and heat-inactivated BBM171 supplemented groups. This result suggests that BBM171 could regulate the balance of immune cells toward the Th1 response. These results are similar to those observed by Wang et al., where oral administration of *Lactobacillus paracasei* L9 improved the PM2.5-induced allergic airway response in mice by rebalancing the Th1/Th2 immune response [28]. Wang et al. also reported that *Bifidobacterium infantis* attenuated allergic airway response by promoting Th1 and inhibiting Th2 immune responses in OVA-induced allergic mice [29]. 

To further analyze the effect of BBM171 on the immunomodulatory response to allergens, splenocytes from OVA-sensitized mice were re-stimulated with OVA and live or heat-activated BBM171 ex vivo. As shown in Figure 6, splenocytes from OVA-sensitized mice showed increased IFN-γ and IL-12 but decreased IL-4, IL-5, and IL-13 production in both live and heat-activated BBM171 treatment groups. In addition, the levels of proinflammatory cytokine IL-17A and regulatory cytokine IL-10 were measured. The level of IL-17A was significantly reduced in the live BBM171 treatment group compared with the OVA group, and a decreasing trend was observed in the heat-inactivated BBM171 group compared with the OVA group. Moreover, IL-10 levels were elevated in live and heat-inactivated BBM171 treatment groups. These results are similar to those of Ren-Long Jan et al., who reported that *L. gasseri* suppresses Th17 proinflammatory response [30]. In addition, an anti-asthmatic effect study by Shabana et al. indicated that vitamin D supplementation reduced serum IL-17A levels and elevated serum IL-10 levels in patients with persistent asthma; hence, the IL-17A/IL-10 ratio could be a predictive biomarker for asthma improvement in patients [31]. Therefore, we speculated that BBM171 improved allergic reactions by regulating the balance between Th1 and Th2 cells by releasing IL-12, which favors the emergence of Th1 cells, in contrast to Th2-driven cytokines such as IL-4 and IL-13. Meanwhile, treatment with BBM171 inhibited the proinflammatory cytokine IL-17A and possessed the T-reg cell-responsive ability to elevate IL-10 production. 

Previous studies have assessed the beneficial effects of probiotics in mouse models of allergic inflammation, and the anti-allergic activity of probiotics is strain-specific [8]. For example, orally administered LGG showed anti-allergic effects in birch pollen-induced or OVA-induced asthma mouse models by inhibiting inflammatory cell infiltration [12,32]. *Lactobacillus casei* HY2782 ameliorated allergic symptoms and reduced IL-4 and IL-5 levels in PM2.5-induced allergic mice [33]. However, studies on the immunomodulatory properties of *L. gasseri* strains are limited and inconsistent. *L. gasseri* SBT2055 protects against influenza virus or respiratory syncytial virus infections by enhancing IgA production or increasing IFN-β or IFN-γ expression. In a study by Li et al., *L. gasseri* strains exerted beneficial effects in an animal model of asthma simulated using house dust mite (HDM) extracts [34]. The results indicated that *L. gasseri* strains limited the inflammatory state expressed by lung cell infiltration and histological scoring while decreasing IgE levels. However, there was no significant difference in Th2-driven cytokines (IL-5 and IL-13) in HDM-treated model mice [34]. Furthermore, to our knowledge, *L. paragasseri* has not been reported to have anti-allergic activity.

In the present study, we reported the anti-allergic effects of live and heat-inactivated forms of *L. paragasseri* BBM171, a strain isolated from breast milk, in Taiwan. According to the Food and Agriculture Organization (FAO)/World Health Organization (WHO), probiotics are beneficial live microorganisms. Favorable properties of heat-inactivated bacteria have been observed in both in vitro and in vivo studies. Compared to live probiotics, heat-inactivated probiotics have advantages in safety and stability [35]. Our results revealed that both live and heat-inactivated BBM171 improved OVA-induced allergic inflammation symptoms by modulating the host immune response to Th1-drive cytokines. As both live and heat-inactivated BBM171 exhibit Th1-dominant immune-stimulatory activity, there must be specific components that resist heat treatment and are recognized by the immune system [36]. The active components of the cell wall, such as peptidoglycans (PGNs), have been considered factors for immunomodulatory activities that induce Th1 cytokines. Sashihara et al. reported a higher correlation between IL-12 (p70) stimulatory activity and the number of PGNs. They further indicated that the presence of abundant amounts of peptidoglycan in heat-inactivated *L. gasseri* OLL2809 promotes IL-12 (p70) production, which in turn may prevent or ameliorate allergic diseases [37,38]. Notably, we observed that heat-inactivated BBM171 was a more potent inducer of Th1 cytokines (IFN-γ and IL-12) than live BBM171 in splenocytes of OVA-allergic mice. This result may be related to the signal transduction induced by bacterial cell surface components, including PGNs, which could be exposed to heat treatment to accelerate immune responses. However, in the present study, both live and heat-inactivated BBM171 were effective in improving allergic symptoms in mice following 48 days of continuous supplementation, and there was no significant difference between them. Thus, both live and heat-inactivated forms of *L. paragasseri* BBM171 are equally effective in improving allergic symptoms when supplemented for longer periods.

Limitations of this study include the use of only female mice in the in vivo experiments, as female mice are more susceptible than male mice to OVA-induced allergic airway inflammation [39,40]. Therefore, future experiments using both male and female mice and human studies may be needed to demonstrate the effectiveness of BBM171. In addition, the effects of BBM171 on the alteration of T lymphocyte subtypes, detected by flow cytometry, and modulation of the gut microbiome should be analyzed to further investigate the action mechanism of BBM171.

## 5. Conclusions

**Figure 7:**
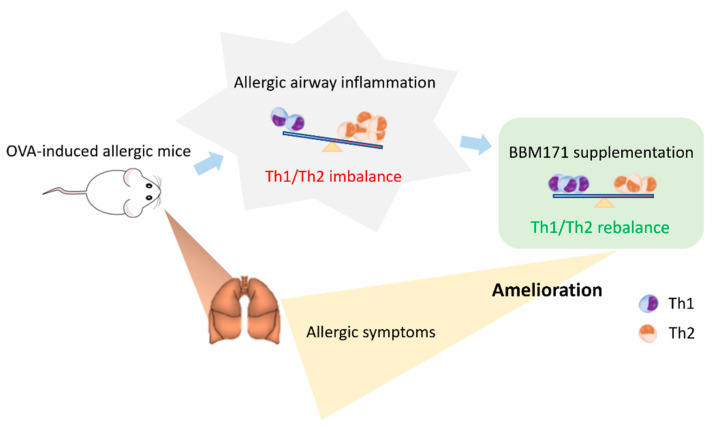
*L. paragasseri* BBM171 alleviated the OVA-induced allergic response by abolishing the accumulation of inflammatory cells in the BALF and maintaining the balance of Th1/Th2.

Taken together, the present study suggests that *L. paragasseri* BBM171 is a novel probiotic that improves allergy-like responses in OVA-sensitized mice (Figure 7). Oral administration of live or heat-inactivated BBM171 significantly reduced allergic serum Ig levels, inflammatory scores, and mucus production in the lungs of OVA-induced mice. Furthermore, BBM171 effectively alleviated allergic responses in OVA-sensitized and OVA-challenged mice by modulating the Th1/Th2 responses toward Th1 dominance. Cultured splenocytes from OVA-sensitized mice also revealed that BBM171 treatment possessed the T-reg cell-responsive ability to elevate IL-10 production. In addition, heat-inactivated BBM171 showed similar anti-allergic potency as live bacteria, indicating their applicability.

## Data Availability

The datasets generated during and/or analyzed during the current study are available from the corresponding author on reasonable request.

## References

[1] Kämpe M., Stolt I., Lampinen M., Janson C., Stålenheim G., Carlson M. (2011). Patients with allergic rhinitis and allergic asthma share the same pattern of eosinophil and neutrophil degranulation after allergen challenge. Clin. Mol. Allergy.

[2] Akdis C.A., Arkwright P.D., Brüggen M.-C., Busse W., Gadina M., Guttman-Yassky E., Kabashima K., Mitamura Y., Vian L., Wu J. (2020). Type 2 immunity in the skin and lungs. Allergy.

[3] Zheng H., Zhang Y., Pan J., Liu N., Qin Y., Qiu L., Liu M., Wang T. (2021). The Role of Type 2 Innate Lymphoid Cells in Allergic Diseases. Front. Immunol..

[4] Zhao J., Lloyd C., Noble A. (2012). Th17 responses in chronic allergic airway inflammation abrogate regulatory T-cell-mediated tolerance and contribute to airway remodeling. Mucosal Immunol..

[5] Yang C., Tian J., Ko W., Shih C., Chiou Y. (2018). Oligo-fucoidan improved unbalance the Th1/Th2 and Treg/Th17 ratios in asthmatic patients: An ex vivo study. Exp. Ther. Med..

[6] Hu Y., Chen Z., Zeng J., Zheng S., Sun L., Zhu L., Liao W. (2020). Th17/Treg imbalance is associated with reduced indoleamine 2,3 dioxygenase activity in childhood allergic asthma. Allergy Asthma Clin. Immunol..

[7] Miranda C., Contente D., Igrejas G., Câmara S.P.A., Dapkevicius M.D.L.E., Poeta P. (2021). Role of Exposure to Lactic Acid Bacteria from Foods of Animal Origin in Human Health. Foods.

[8] Jakubczyk D., Górska S. (2021). Impact of Probiotic Bacteria on Respiratory Allergy Disorders. Front. Microbiol..

[9] Maldonado Galdeano C., Cazorla S.I., Lemme Dumit J.M., Vélez E., Perdigón G. (2019). Beneficial Effects of Probiotic Consumption on the Immune System. Ann. Nutr. Metab..

[10] Ren D., Wang D., Liu H., Shen M., Yu H. (2019). Two strains of probiotic *Lactobacillus* enhance immune response and promote naive T cell polarization to Th1. Food Agric. Immunol..

[11] Eslami M., Bahar A., Keikha M., Karbalaei M., Kobyliak N., Yousefi B. (2020). Probiotics function and modulation of the immune system in allergic diseases. Allergol. Immunopathol..

[12] Spacova I., Petrova M.I., Fremau A., Pollaris L., Vanoirbeek J., Ceuppens J.L., Seys S., Lebeer S. (2018). Intranasal administration of probiotic *Lactobacillus rhamnosus* GG prevents birch pollen-induced allergic asthma in a murine model. Allergy.

[13] Lyons K.E., Ryan C.A., Dempsey E.M., Ross R.P., Stanton C. (2020). Breast Milk, a Source of Beneficial Microbes and Associated Benefits for Infant Health. Nutrients.

[14] Nuzzi G., Di Cicco M., Peroni D. (2021). Breastfeeding and Allergic Diseases: What’s New?. Children.

[15] Pérez-Cano F.J., Dong H., Yaqoob P. (2010). In vitro immunomodulatory activity of Lactobacillus fermentum CECT5716 and Lactobacillus salivarius CECT5713: Two probiotic strains isolated from human breast milk. Immunobiology.

[16] Rodríguez-Sojo M., Ruiz-Malagón A., Rodríguez-Cabezas M., Gálvez J., Rodríguez-Nogales A. (2021). *Limosilactobacillus fermentum* CECT5716: Mechanisms and Therapeutic Insights. Nutrients.

[17] Tanizawa Y., Tada I., Kobayashi H., Endo A., Maeno S., Toyoda A., Arita M., Nakamura Y., Sakamoto M., Ohkuma M. (2018). *Lactobacillus paragasseri* sp. nov., a sister taxon of *Lactobacillus gasseri*, based on whole-genome sequence analyses. Int. J. Syst. Evol. Microbiol..

[18] Itoh H., Uchida M., Sashihara T., Ji Z.-S., Li J., Tang Q., Ni S., Song L., Kaminogawa S. (2010). *Lactobacillus gasseri* OLL2809 is effective especially on the menstrual pain and dysmenorrhea in endometriosis patients: Randomized, double-blind, placebo-controlled study. Cytotechnology.

[19] Dang Y., Reinhardt J.D., Zhou X., Zhang G. (2014). The Effect of Probiotics Supplementation on Helicobacter pylori Eradication Rates and Side Effects during Eradication Therapy: A Meta-Analysis. PLoS ONE.

[20] Liu Y.-W., Liao T.-W., Chen Y.-H., Chiang Y.-C., Tsai Y.-C. (2014). Oral Administration of Heat-Inactivated Lactobacillus plantarum K37 Modulated Airway Hyperresponsiveness in Ovalbumin-Sensitized BALB/c Mice. PLoS ONE.

[21] Jiang X.-H., Li C.-Q., Feng G.-Y., Luo M.-J., Sun Q.-X. (2020). Inhalation of nebulized Mycobacterium vaccae can protect against allergic bronchial asthma in mice by regulating the TGF-β/Smad signal transduction pathway. Allergy Asthma Clin. Immunol..

[22] Roldán N.G., Duda K.A. (2020). Editorial: Role of Lipids in the Dynamics of Allergic Airway Inflammation. Front. Immunol..

[23] Dharmage S., Perret J.L., Custovic A. (2019). Epidemiology of Asthma in Children and Adults. Front. Pediatr..

[24] Gorczynski R.M., Maqbool T., Hoffmann G. (2019). Mechanism(s) of prolonged attenuation of allergic responses after modulation of idiotypic regulatory network. Allergy Asthma Clin. Immunol..

[25] Ansotegui I.J., Melioli G., Canonica G.W., Caraballo L., Villa E., Ebisawa M., Passalacqua G., Savi E., Ebo D., Gómez R.M. (2020). IgE allergy diagnostics and other relevant tests in allergy, a World Allergy Organization position paper. World Allergy Organ. J..

[26] Hsu C.-Y., Leu S.-J., Chiang B.-L., E Liu H., Su H.-C., Lee Y.-L. (2010). Cytokine gene-modulated dendritic cells protect against allergic airway inflammation by inducing IL-10+IFN-γ+CD4+ T cells. Gene Ther..

[27] Chung F. (2001). Anti-inflammatory cytokines in asthma and allergy: Interleukin-10, interleukin-12, interferon-γ. Mediat. Inflamm..

[28] Wang X., Hui Y., Zhao L., Hao Y., Guo H., Ren F. (2017). Oral administration of Lactobacillus paracasei L9 attenuates PM2.5-induced enhancement of airway hyperresponsiveness and allergic airway response in murine model of asthma. PLoS ONE.

[29] Wang W., Luo X., Zhang Q., He X., Zhang Z., Wang X. (2020). Bifidobacterium infantis Relieves Allergic Asthma in Mice by Regulating Th1/Th2. Med. Sci. Monit..

[30] Jan R.-L., Yeh K.-C., Hsieh M.-H., Lin Y.-L., Kao H.-F., Li P.-H., Chang Y.-S., Wang J.-Y. (2011). *Lactobacillus gasseri* suppresses Th17 pro-inflammatory response and attenuates allergen-induced airway inflammation in a mouse model of allergic asthma. Br. J. Nutr..

[31] Shabana M.A., Esawy M., Ismail N.A., Said A.M. (2019). Predictive role of IL-17A/IL-10 ratio in persistent asthmatic patients on vitamin D supplement. Immunobiology.

[32] Wu C.-T., Chen P.-J., Lee Y.-T., Ko J.-L., Lue K.-H. (2016). Effects of immunomodulatory supplementation with Lactobacillus rhamnosus on airway inflammation in a mouse asthma model. J. Microbiol. Immunol. Infect..

[33] Nam W., Kim H., Bae C., Kim J., Nam B., Lee Y., Kim J., Park S., Lee J., Sim J. (2020). *Lactobacillus* HY2782 and *Bifidobacterium* HY8002 Decrease Airway Hyperresponsiveness Induced by Chronic PM2.5 Inhalation in Mice. J. Med. Food.

[34] Li L., Fang Z., Liu X., Hu W., Lu W., Lee Y.-K., Zhao J., Zhang H., Chen W. (2020). Lactobacillus reuteri attenuated allergic inflammation induced by HDM in the mouse and modulated gut microbes. PLoS ONE.

[35] Piqué N., Berlanga M., Miñana-Galbis D. (2019). Health Benefits of Heat-Killed (Tyndallized) Probiotics: An Overview. Int. J. Mol. Sci..

[36] Mei H.-C., Liu Y.-W., Chiang Y.-C., Chao S.-H., Mei N.-W., Liu Y.-W., Tsai Y.-C. (2013). Immunomodulatory Activity ofLactococcus lactisA17 from Taiwan Fermented Cabbage in OVA-Sensitized BALB/c Mice. Evid.-Based Complement. Altern. Med..

[37] Sashihara T., Sueki N., Ikegami S. (2006). An Analysis of the Effectiveness of Heat-Killed Lactic Acid Bacteria in Alleviating Allergic Diseases. J. Dairy Sci..

[38] Onishi K., Mochizuki J., Sato A., Goto A., Sashihara T. (2020). Total RNA and genomic DNA of Lactobacillus gasseri OLL2809 induce interleukin-12 production in the mouse macrophage cell line J774.1 via toll-like receptors 7 and 9. BMC Microbiol..

[39] Takeda M., Tanabe M., Ito W., Ueki S., Konnno Y., Chihara M., Itoga M., Kobayashi Y., Moritoki Y., Kayaba H. (2013). Gender difference in allergic airway remodelling and immunoglobulin production in mouse model of asthma. Respirology.

[40] Melgert B.N., Postma D.S., Kuipers I., Geerlings M., Luinge M.A., van der Strate B.W.A., Kerstjens H.A.M., Timens W., Hylkema M.N. (2005). Female mice are more susceptible to the development of allergic airway inflammation than male mice. Clin. Exp. Allergy.

